# Post-partum spontaneous coronary artery dissection refractory to conservative management

**DOI:** 10.21542/gcsp.2020.34

**Published:** 2020-12-31

**Authors:** Eric Etchill, Nicholas Clarke, Katherine Giuliano, Jennifer Lawton, Chun Woo Choi

**Affiliations:** 1 Division of Cardiac Surgery, Department of Surgery, Johns Hopkins University School of Medicine, Baltimore, Maryland, USA

## Abstract

This case report describes a 31-year-old female who was diagnosed with a spontaneous coronary artery dissection (SCAD) of her left anterior descending artery during the post-partum period. She failed nonoperative management and was found to have extensive propagation of the dissected vessel associated with recurrent chest pain. She ultimately underwent coronary artery bypass grafting which restored perfusion and cardiac wall motion. A brief discussion of the presentation, diagnosis, and management of SCAD follows.

## Case report

We present a 31-year-old female patient (gravida five, para two, abortus three) who presented to the emergency department with acute onset severe chest pain nine days after vaginal delivery following an uneventful pregnancy with routine prenatal care. The pain, described as substernal and a squeezing, lasted for several minutes before resolution with rest. Her past medical history was notable for obesity, anxiety, bipolar disorder, a ten pack-year smoking history (quit eight months prior), and marijuana use (last use eight months prior).

Her past surgical history was remarkable for two remote episodes of cervical dilation and curettage and a right salpingectomy for a ruptured ectopic pregnancy complicated by significant blood loss three years prior. Her family history was unremarkable. She was on no medications aside from prenatal vitamins, denied any recent alcohol, tobacco, or drug use, and reported being allergic to ibuprofen.

**Figure 1.**
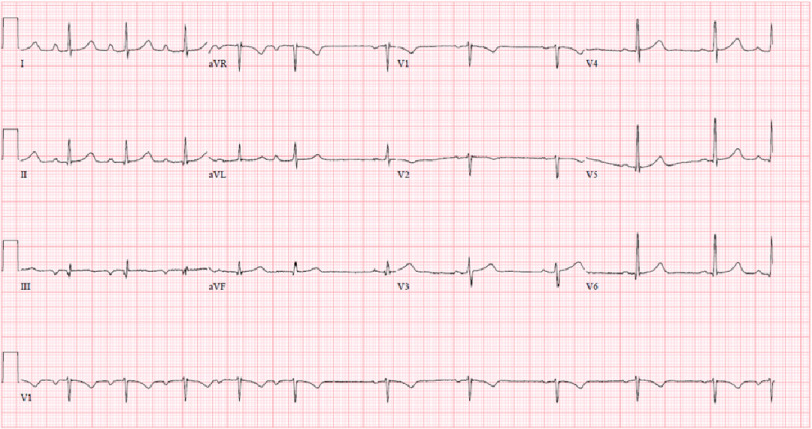
EKG shortly after symptom onset demonstrating sinus rhythm with a Mobitz type 1 block.

**Figure 2.**
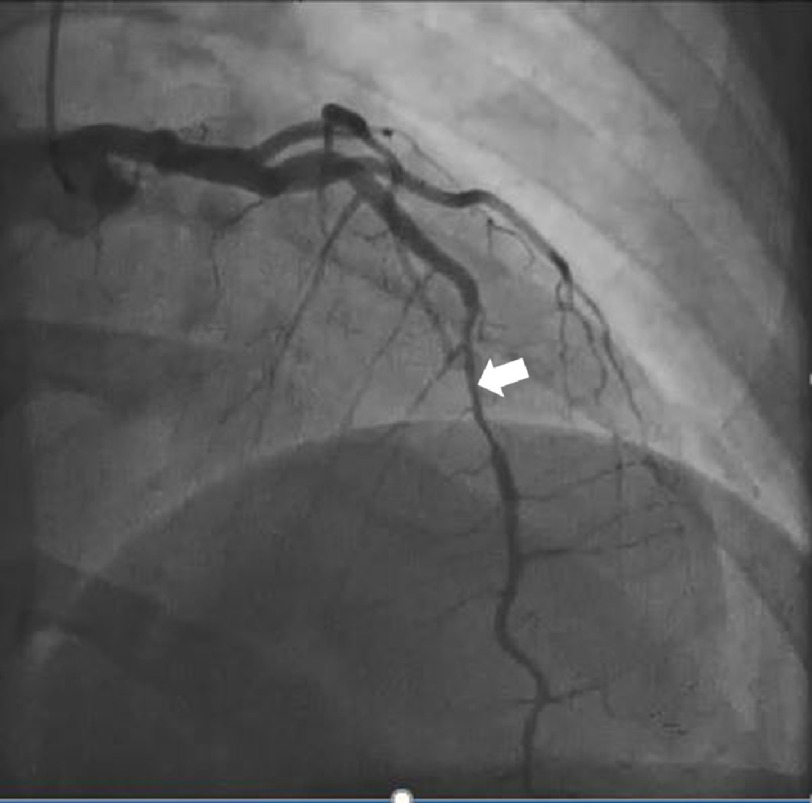
Initial cardiac catheterization demonstrating 70–80% stenosis of the mid left anterior descending artery consistent with spontaneous coronary artery dissection (arrow).

At the time she presented to the emergency department she was afebrile, normotensive, heart rate 70 beats per minute, and eupneic. Her physical examination was unremarkable. She was admitted to the obstetrics service where her troponin was found to be elevated at 0.41, which increased to 1.19 five hours later. Her electrocardiogram (EKG) was notable for a sinus rhythm with a Mobitz type 1 block (Figure 1). Cardiology was consulted and recommended 325 mg of acetylsalicylic acid (ASA), a transthoracic echocardiogram (TTE), and cardiac catheterization. Her TTE was unremarkable, with an ejection fraction of 60–65% and normal diastolic filling. Cardiac catheterization demonstrated a long segment of 70–80% stenosis in the mid left anterior descending artery (LAD) with TIMI 3 flow distally, consistent with spontaneous coronary artery dissection (SCAD) (Figure 2). The remainder of the vessels were otherwise angiographically normal. She was diagnosed with a non-ST-elevation myocardial infarction (NSTEMI) secondary to SCAD and prescribed aspirin with plans for discharge.

**Figure 3.**
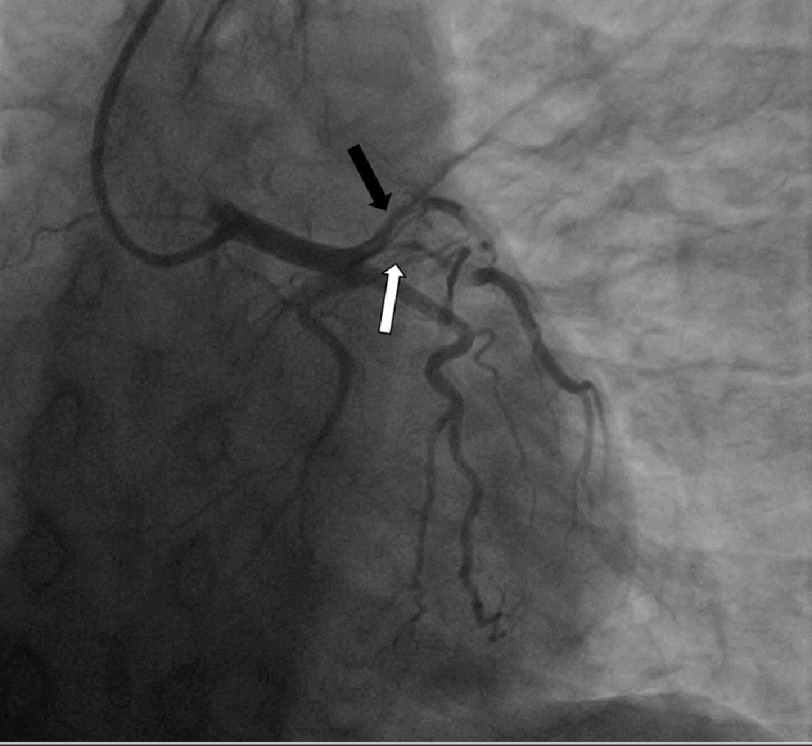
Emergent repeat cardiac catheterization demonstrating extensive propagation of the dissection proximally from the prior mid left anterior descending artery site (black arrow), and to the proximal portion of a large high diagonal branch (white arrow).

Prior to discharge the following day she developed recurrent acute chest pain. An EKG revealed new ST-segment elevations in leads I and aVL, and a subsequent EKG demonstrated new ST-segment elevations in leads V1 and V2. She was transferred to the cardiac critical care unit for observation. A bedside TTE demonstrated new extensive anterolateral hypokinesis compared to prior, with a large myocardial territory at risk. An emergent repeat cardiac catheterization demonstrated extensive propagation of the SCAD proximally from the prior mid-LAD site back to the ostium, and to the proximal portion of a large high diagonal branch. There was now TIMI 0.5–1 flow to the apical LAD (Figure 3). The left main, left circumflex, and right coronary arteries were unaffected.

The patient was then emergently referred for coronary artery bypass grafting (CABG) given the prohibitive risk of percutaneous coronary intervention due to high risk of further propagating the dissection, and the length of stent that would be required likely occluding a large diagonal branch. She underwent coronary artery bypass grafting with the left internal mammary artery (LIMA) to the LAD and a saphenous vein graft (SVG) from the aorta to the first diagonal artery. Anteroseptal wall motion on transesophageal echocardiogram (TEE) was significantly improved at the conclusion of the case. She was extubated in the intensive care unit (ICU) hours after arrival. Over the next several days she was weaned off inotropic support and was discharged postoperative day six with plans for cardiology follow up and screening for fibromuscular dysplasia.

## Discussion

**Figure 4.**
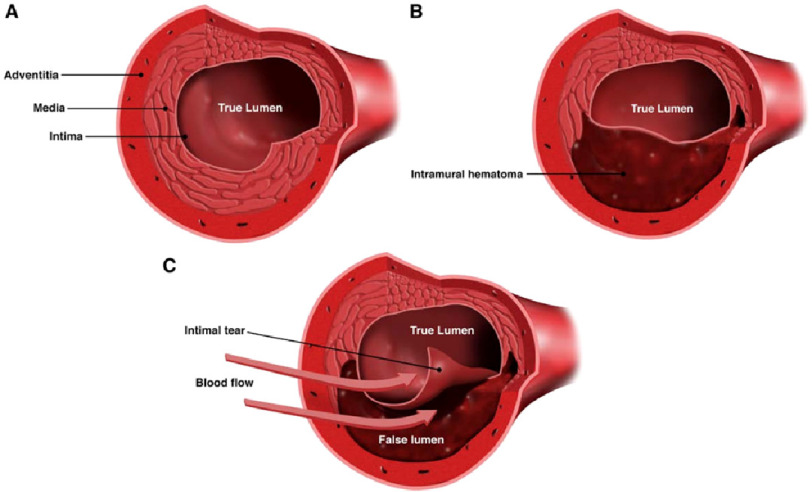
Cross-sectional views of the coronary artery. (A) Normal coronary artery. (B) Coronary artery with intramural hematoma. C, Coronary artery with intimal tear. Spontaneous coronary artery dissection is characterized by the spontaneous formation of an intramural hematoma, which can lead to compression of the true lumen and myocardial infarction. An intimal tear may be present. Created by and used with permission from Dominic Doyle, MA.

Spontaneous coronary artery dissection (SCAD) is an underdiagnosed acute coronary syndrome that occurs in otherwise normal coronary arteries.^1^ While poorly studied, SCAD may result in myocardial ischemia, myocardial infarction, ventricular arrhythmias, and sudden cardiac death.^1^ There are two proposed theories to explain the mechanism of SCAD. The first is the intimal tear hypothesis, in which a primary disruption in the intimal-lumen interface allows for intramural hematoma accumulation inside the false lumen, resulting in separation of the arterial wall (Figure 4).^2^ The second proposed mechanism involves primary coronary arterial wall hemorrhage, possibly due to spontaneous rupture from increased density of the vasa vasorum.^3^

SCAD is more common in younger females and predisposing factors include: atherosclerotic disease, fibromuscular dysplasia, vasospasm, pregnancy and the peripartum period, multiparity, connective tissue disorder, and systemic inflammatory disease.^1,4–6^ Inciting causes include intense exercise, emotional stress, Valsalva maneuvers, recreational drugs, hormone therapy, steroid use, and labor and delivery. In the patient presented, it is plausible that high progesterone levels during pregnancy weakened the media layer of the arterial wall. This weakened arterial wall, combined with a hormone-induced hypercoagulable state, could have led to SCAD with the ensuing vessel thrombosis.^7,8^ Because this is a frequently misdiagnosed and underdiagnosed entity, suspicion for SCAD should be high in individuals with predisposing risk factors and symptoms of coronary insufficiency who otherwise appear low risk for an acute coronary syndrome.^1^

The optimal treatment of SCAD is unclear and ranges from conservative therapy to emergency revascularization with percutaneous coronary intervention (PCI) or coronary artery bypass grafting.^9,10^ Patients who present with acute myocardial infarction (AMI) and have either left main dissection or symptoms of continued ischemia or hemodynamic instability should be referred for emergent revascularization, as occurred in the patient presented.^10–12^

**Figure 5.**
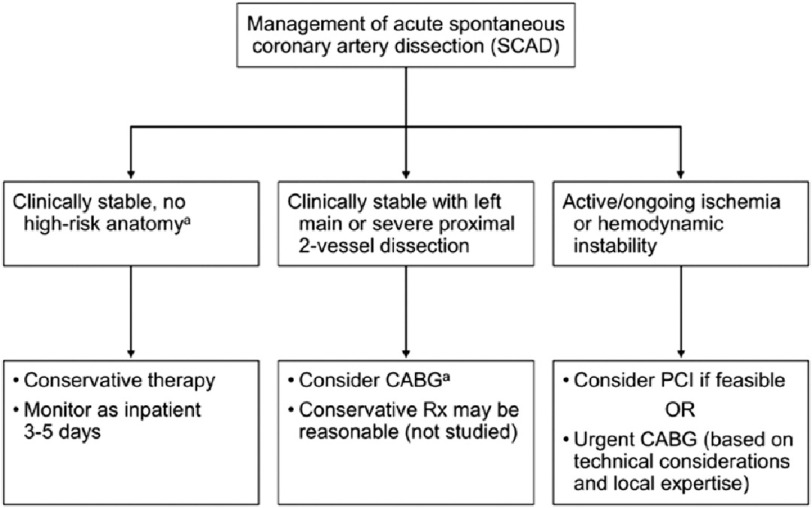
Algorithm for management of acute spontaneous coronary artery dissection. CABG indicates coronary artery bypass grafting; PCI, percutaneous coronary intervention; and Rx, management. ^a^Left main or proximal 2-vessel coronary artery dissection. Reprinted with permission from Hayes et al.^[2]^

In stable patients without the above mentioned features, long-term management with aspirin, beta blockers, and one year of dual antiplatelet therapy has been shown to lower the risk of recurrent SCAD (Figure 5).^2^ Prospective randomized studies are needed to further elucidate the optimal treatment modality in patients with SCAD, particularly those in the peripartum period.^11,13^
